# Impact of Real and Simulated Flights on Psychophysiological Response of Military Pilots

**DOI:** 10.3390/ijerph18020787

**Published:** 2021-01-18

**Authors:** Juan Pedro Fuentes-García, Vicente J. Clemente-Suárez, Miguel Ángel Marazuela-Martínez, José F. Tornero-Aguilera, Santos Villafaina

**Affiliations:** 1 Faculty of Sport Science, University of Extremadura, Avda. Universidad S/N, 10003 Cáceres, Spain; jpfuent@unex.es; 2 Faculty of Sport Sciences, Universidad Europea de Madrid, 28670 Villaviciosa de Odón, Spain; josefrancisco.tornero@universidadeuropea.es; 3 Grupo de Investigación en Cultura, Educación y Sociedad, Universidad de la Costa, 080002 Barranquilla, Colombia; 4 Base Aérea Talavera la Real, ALA-23 Grupo Fuerza Aérea, 06071 Talavera la Real, Spain; mamatm@ea.mde.es; 5 Physical Activity and Quality of Life Research Group (AFYCAV), Faculty of Sport Science, University of Extremadura, 10003 Cáceres, Spain; svillafaina@unex.es

**Keywords:** HRV, army, simulator, flight, anxiety, perceived exertion

## Abstract

Objective: The present research aimed to analyse the autonomic, anxiety, perceived exertion, and self-confidence response during real and simulated flights. Methods: This cross-sectional study participated 12 experienced male pilots (age = 33.08 (5.21)) from the Spanish Air Force. Participants had to complete a real and a simulated flight mission randomly. The heart rate variability (HRV), anxiety, self-confidence, and rating of perceived exertion were collected before and after both manoeuvres, and HRV was also collected during both simulated and real flights. Results: When studying the acute effects of real and simulated flights, the mean heart rate, the R-to-R interval, the cognitive anxiety and the perceived exertion were significantly impacted only by real flights. Furthermore, significant differences in the mean heart rate and RR interval were found when compared to the acute effects of real and simulated flights (with higher acute effects observed in real flights). Additionally, when compared the HRV values during simulated and real flights, significant differences were observed in the RR and heart rate mean (with lower RR interval and higher heart rate mean observed during real flights). Conclusion: Real flights significantly reduced the RR interval and cognitive anxiety while increased the heart rate mean and the rating of perceived exertion, whereas simulated flights did not induce any significant change in the autonomic modulation.

## 1. Introduction

Flight simulators have been used in aviation as an economical and safety tool to train pilots, providing a simulated environment which could mimic real conditions [1]. In order to study the transfer between training conditions and aircraft [2], previous studies have used different psychophysiological tools to investigate the cognitive demands of both simulated and real flights [3,4]. This is relevant since this operation requires higher cognitive demands [2,5,6,7] and, therefore, the evaluation of the mental workload has emerged as a cornerstone.

Heart rate variability (HRV) is a non-invasive tool which studies the successive heartbeats variation [8]. This evaluates the balance between sympathetic and parasympathetic nervous systems, as well as it has been considered a measure of heart-brain interaction since it could be modified by cognitive, attentional, anxiogenic, or physical stimulus [9,10,11]. Thus, when the activity of the sympathetic nervous system predominates, the HRV is reduced. Instead, when parasympathetic activity increases, the HRV is higher. For these reasons, the HRV is considered as a cognitive load biomarker [11,12,13].

HRV measures have been performed in military pilots to study the sympathetic activity during flights [14,15]. In this regard, Sauvet, et al. [16] showed that flights induced a progressive decrease of RR intervals, increasing the sympathetic activity. The sympathetic activation could induce increased anaerobic metabolism or increased anxiety and stress perceptions among other psychophysiological effects [17]. Autonomic modulation analysis was used in a military population as a stress marker, showing how different military manoeuvres, independently of land or flight units, produced an increased sympathetic modulation [18]. Moreover, the fatigue induced by flights can also be detected by spirometry, handgrip strength, and stress and exertion rates as previous authors showed in this special population [19,20,21,22].

The development of flight simulators has allowed its use as a way of training, avoiding high-risk conditions with catastrophic consequences. Due to this fact, as well as to economise training (since real flights have enormous costs), the number of real flights has decreased whereas the number of hours in the flight simulator increased. However, the psychophysiological response of these conditions (real and simulated flights) and the comparison between real and simulated flight condition have been poorly studied. Therefore, the aims of the present study were: (1) To analyse the acute effects of a simulated and a real flight in the autonomic modulation, anxiety, perceived exertion, and self-confidence; and (2) to compare the autonomic modulation during a real and a simulated flight. Since flight simulator cannot mimic the physical demands of a real flight, our hypotheses are: (1) The impact of acute effects (on HRV and anxiety) will be higher in the real than in the simulator; and (2) the HRV will be lower during a real flight than during a simulated flight.

## 2. Materials and Methods

### 2.1. Participants

Twelve experienced military pilots participate in this cross-sectional study. Pilots had a mean age of 33.08 (5.21) years and an experience of 13.25 (5.15) years of military service. Procedures were approved by the university ethics committee (approval number: 206/2019) as well as pilots agreed to participate in this study, giving written consent. Table 1 shows pilots’ characteristics.

### 2.2. Procedure

Participants were evaluated before, during and after two flights: (1) A real flight with a F5 aircraft; and (2) a simulated flight with an operational F-5 M (Indra Company, Madrid, Spain) flight simulator. Each protocol, real or simulated flights, were performed on consecutive separate days. The order between simulated and real flights was randomised. The mission, both simulated and real, were the same with: (1) An individual takeoff; (2) G-warm up/G- awareness below FL 180; (3) air–air mission with two set-ups; (4) air–ground attack on the selected target, and (5) landing without reduction of visibility. Both real and flight protocols lasted 45 min. For analysis purposes, the whole protocol duration (with all the manoeuvres) was used to study the HRV during real and simulated flights.

HRV, anxiety, perceived exertion, and self-confidence were assessed immediately after and before real and simulated missions. The HRV baseline was recorded once starting the protocol. Moreover, the HRV was assessed during both real and simulated flight mission. In order to avoid potential confounding factors, participants were asked to not take alcohol, coffee, or caffeinated drinks 24-hours before undergoing the protocol, since their consumption could affect the nervous system, and, therefore, HRV variables. None of the participants had smoking habits or took cardioactive medication such as antidepressant, antipsychotic, or antihypertensive medication [23].

### 2.3. HRV Acquisition and Preprocessing Steps

The HRV was recorded using a reliable heart rate monitor (Polar RS800CX, Oy, Kempele, Finland) [24] and analysed with the Kubios HRV software (v. 3.3) [25]. The Task Force’s recommendations of the European Society of Cardiology and the North American Society of Pacing and Electrophysiology [26] were followed in this study. Thus, the baseline measure lasted 5 min (which is considered a short-term record). RR data recorded by the heart rate monitor was exported to the Kubios HRV software where different preprocessing steps were applied. In this regard, a middle filter was applied to correct possible artefacts. Those RR intervals which are shorter/longer than 0.25 s, compared to previous beats average were automatically identified, as well as they were replaced by cubic spline interpolation [27,28].

### 2.4. Outcomes

#### 2.4.1. HRV

Time, frequency and non-linear variables were calculated using the Kubios HRV software. In the time domain, the mean heart rate (mean HR), RR intervals, RR50 count divided by the total number of all RR ranges (Pnn50), and the square root of differences between adjacent RR intervals (RMSSD) were extracted. In the frequency domain, the low frequency (LF, 0.04–0.15 Hz) and high frequency (HF, 0.15–0.4 Hz) ratio (LF/HF) and Total Power were included. The non-linear measures, such as RR variability from heartbeat to short term Poincaré graph (width) (SD1) and RR variability from heartbeat to long term Poincaré graph (length) (SD2). Further information regarding HRV variables can be found in the following articles [9,29].

#### 2.4.2. Anxiety Measurements

The Competitive State Anxiety Inventory-2R (CSAI-2R) (Spanish version) was used to assess the pre-competitive anxiety of the participants [30,31]. This questionnaire has 17 items, where cognitive anxiety, somatic anxiety and self-confidence can be extracted. Moreover, anxiety was also measured by the State-Trait Anxiety Inventory (STAI-E) (Spielberger et al., 1971), which consisted of 20 items and where the participants reported their state of anxiety at that time. The score range for the test is 20–80, indicating a higher level of anxiety a higher score [32]. Additionally, the perceived exertion was assessed by the rating of perceived exertion (RPE) 6–20 scale [33].

### 2.5. Statistical Analysis

The SPSS statistical package (version 20.0; SPSS, Inc., Chicago, IL, USA) was used to analyse the data. Following the results of a Shapiro–Wilk test, non-parametric tests were employed.

The Wilcoxon signed-rank test was used to examine the difference between the pre- and post-measures for each variable. Moreover, in order to compare the impact of real vs. simulated flight, the post-values were normalised (by subtracting the baseline). Effect sizes [r] were calculated for the non-parametric tests, classified as follows: 0.5 is a large effect, 0.3 is a medium effect and 0.1 is a small effect [34,35].

## 3. Results

### 3.1. HRV and Perceived Anxiety and Self-Confidence before and after a Real and a Simulated Flight

Table 2 shows the acute effects of a real vs. a simulated flight mission on the HRV of experienced pilots. Significant results were obtained in the mean HR and RR variables (*p*-value < 0.05) when compared the baseline and the post-measure after a real flight. Regarding simulated flight, significant differences were not found between baseline and post-measure. Moreover, significant differences were obtained when compared to real vs. simulated flight. In this regard, significant differences were found in mean HR and RR, indicating higher mean HR and lower RR intervals after a real flight.

Table 3 shows the acute effects of a real and a simulated flight mission on the rating of perceived exertion, anxiety, and self-confidence of experienced military pilots. In this regard, differences between baseline and post-measure were found in the rating of perceived exertion (*p*-value = 0.010) and the cognitive anxiety (*p*-value 0.024), showing an increased rating of perceived exertion and lower cognitive anxiety after a real flight. Regarding differences between real and simulated flight, statistically, and significant differences were not achieved by any of the studied variables.

### 3.2. HRV during a Real and a Simulated Flight

Table 4 shows the HRV during simulated and real flights. Results showed significant differences between real and simulated flights, exhibiting higher mean HR and lower RR interval during a real flight.

## 4. Discussion

This study aimed to analyse the autonomic, anxiety, perceived exertion, and self-confidence response during real and simulated flight. Regarding the first hypothesis, “the impact of acute effects (on HRV and anxiety) will be higher in the real than in the simulator”, cannot be accepted since not all the variables reached the significance level (*p*-value < 0.05). Only significant differences (between baseline and post measures) were found in the mean HR, RR interval, cognitive anxiety and perceived exertion in the real flight. Moreover, significant differences in the acute effects were observed in the mean HR and RR intervals between real and simulated flights (with significantly higher values of mean HR and lower RR interval values after the real flight). Regarding the second hypothesis, “the HRV will be lower during a real flight than during a simulated flight” cannot be totally accepted since only mean HR and RR interval showed significant differences between real and simulated flights. Higher values of mean HR and lower values of RR interval were observed during real flights.

These results highlighted the importance of flight simulator for training purposes in pilots. Not all the expected differences were reached, which could mean that planning and task design of the simulated task are close to the real condition. However, since the simulator cannot mimic the G forces or vibration, the significant differences which can be observed in the RR interval, mean HR, and perceived exertion can be derived from the physical demands of real flights. Furthermore, the differences in the cognitive anxiety observed before a real and simulated flight can be due to the responsibility and risk derived from real flights. Therefore, in order to totally mimic real conditions, future flight simulators should incorporate immersive virtual reality technology simulating G forces and vibration. Additionally, the relevant information that provides the HRV (in both cognitive and physical spheres) could be used to design more individualised training controlling the training load and therefore increasing the efficiency and the performance of pilots during flights.

The study of the HRV informed about the balance between parasympathetic and sympathetic nervous systems. Previous studies in aviation field showed how simulated and real flights could reduce the HRV [3,5,15,21]. This is relevant due to the negative impact of increased sympathetic activity that produces a reduction in memory and decision making processes [36,37]. However, our results showed that HR mean and RR intervals were negatively impacted by real flights while other time domain, frequency domain or non-linear measures were not. Since the mean heart rate was below 100 beats/min, it would suggest that these differences were due to a parasympathetic activity reduction rather than an increase in sympathetic activity. A previous study that analyses the effect of defence and attacks air combat manoeuvres on air combat fighter pilots’ psychophysiological response did not detect significant differences between pre versus post or pre versus during flight [15]. Authors stated that this behaviour could be due behaviours’ anticipatory anxiety response which started before the flight manoeuvres [15]. Nevertheless, in our study, statistically significant differences were not observed in the HRV when compared real vs. simulator or even pre vs. post (for both real and simulator conditions).

In this regard, previous studies have highlighted the usefulness, due to the high sensitivity, of this variable (RMSSD) to measure the autonomic modulation [21,38]. However, our results did not show any significant effect on this variable. Hypothetically, these results could also be explained since all the analysed pilots were expert. A previous study reinforces this explanation since lower HRV values were related to a lower level of experience [20]. Authors explained that this could be an adaptive response to this stressful environment [20]. Another study showed that a pilot’s first flight was the most stressful [7,39]. Thus, novice pilots might exhibit higher sympathetic, reducing the HRV. Therefore, future studies should focus on comparing the different level of expertise in pilots.

Previous studies have investigated the impact of flights on the anxiety and rating of perceived exertion. Regarding anxiety, it was shown [21] that anxiety was higher before flight missions, highlighting the anticipatory anxiety response of pilots. This was in line with our results when higher cognitive anxiety was found before a real flight which could be due to the responsibility of flight a real aircraft. This is congruent with the HRV results where differences were not obtained (significant differences were not achieved between pre and post or during flight assessments), and an anticipatory response emerged as a possible explanation. Moreover, regarding the rating of perceived exertion, real flight exhibited higher values than simulated one. This could be due to the stress and anxiogenic response induced by mechanical load such as vibration or G forces which pilots have to suffer during a real flight. This is also supported by the acute effects observed on the autonomic modulation where significant differences were reported on RR and heart rate mean.

This study has some limitations which should be highlighted. First, the sample size was small, so probably only large differences have reached the statistical significance level. Second, the sample was composed by experienced pilots which mean that results cannot be extrapolated to novice pilots. Third, the SPSS package uses z-ratio, which is applicable for at least ten observations. Although this study has twelve observations, results from Wilcoxon signed-rank test could be affected by this issue. Fourth, the breathing rate was not controlled, so the respiratory sinus arrhythmia can impact RR intervals. Lastly, the simulator did not mimic all the real conditions such as vibration, G forces or even oxygen deprivation. Thus, flight simulator could be improved, incorporating this to the simulation in order to make more realistic training for pilots.

## 5. Conclusions

Real flights significantly reduced the RR interval and cognitive anxiety while increased the heart rate mean and the rating of perceived exertion whereas simulated flights did not induce any significant change in the autonomic modulation

## Figures and Tables

**Table 1:** Characteristics of military pilots.

Variable	Mean (SD)
Age (years)	33.08 (5.21)
Military service (years)	13.25 (5.15)
Fear to an accident(0–100)	28.33 (25.17)

**Table 2:** Acute effects of a real vs. a simulated flight mission on heart rate variability (HRV).

Flight Conditions		Baseline	Post	Baseline vs. Post Measure		Acute Effects of a Real vs. a Simulated Mission	
	Variables	Mean (SD)	Mean (SD)	*p*-Value	Effect Size	*p*-Value	Effect Size
Real F.	Mean HR	69.82 (10.70)	98.06 (11.89)	0.003 *	0.847	0.005 *	0.809
Simulated F.			74.56 (14.39)	0.155	0.411		
Real F.	RR	885.54 (136.14)	631.91 (77.11)	0.003 *	0.847	0.005 *	0.809
Simulated F.			837.70 (144.35)	0.155	0.411		
Real F.	Pnn50	19.34 (17.18)	8.65 (8.28)	0.062	0.539	0.139	0.426
Simulated F.			16.04 (12.08)	0.929	0.026		
Real F.	RMSSD	44.28 (25.96)	27.77 (13.50)	0.062	0.539	0.139	0.426
Simulated F.			37.01 (14.39)	0.424	0.231		
Real F.	SDNN	51.05 (27.29)	41.48 (13.88)	0.477	0.205	0.241	0.338
Simulated F.			45.30 (11.91)	0.594	0.154		
Real F.	HF	35.49 (18.01)	24.43 (12.12)	0.155	0.411	0.721	0.103
Simulated F.			28.76 (16.45)	0.075	0.513		
Real F.	LF	64.44 (18.00)	75.51 (12.14)	0.155	0.411	0.721	0.103
Simulated F.			71.17 (16.47)	0.075	0.513		
Real F.	LF/HF	2.95 (2.94)	4.20 (3.14)	0.213	0.359	0.508	0.191
Simulated F.			3.69 (2.65)	0.328	0.282		
Real F.	Total Power	2931.89 (3830.04)	1953.40 (1200.12)	0.859	0.051	0.508	0.191
Simulated F.			2090.28 (1078.28)	0.286	0.308		
Real F.	SD1	31.37 (18.42)	19.64 (9.54)	0.062	0.539	0.139	0.427
Simulated F.			26.18 (10.18)	0.424	0.231		
Real F.	SD2	64.54 (34.65)	55.04 (17.79)	0.594	0.154	0.285	0.309
Simulated F.			58.09 (14.66)	0.534	0.179		

* *p*-value < 0.05. F: Flight; HR: Heart rate; RR: Time between intervals R-R; pNN50: Percentage of intervals >50 ms different from the previous interval; RMSSD: The square root of the mean of the squares of the successive differences of the interval RR; LF/HF: Low frequency (LF) ratio (ms2)/High frequency (HF) (ms2); Total power: The sum of all the spectra; SDNN: Standard deviation of normal-to-normal intervals; SD1: Dispersion, standard deviation, of points perpendicular to the axis of line-of-identity in the Poincaré plot; SD2: Dispersion, standard deviation, of points along the axis of line-of-identity in the Poincaré plot.

**Table 3:** Acute effects of a real vs. a simulated flight mission on the rating of perceived exertion, anxiety and self-confidence.

Flight Conditions		Baseline	Post	Baseline vs. Post-Measure		Acute Effects of a Real vs. a Simulated Mission	
	Variables	Mean (SD)	Mean (SD)	*p*-Value	Effect Size	*p*-Value	Effect Size
Real F.	RPE	8.42 (1.88)	11.17 (2.33)	0.010 *	0.742	0.218	0.355
Simulated F.		7.92 (2.23)	8.82 (2.64)	0.089	0.491		
Real F.	STAI-E	27.42 (8.26)	28.17 (9.37)	0.574	0.162	0.633	0.138
Simulated F.		24.82 (3.87)	24.75 (4.55)	0.459	0.213		
Real F.	Cognitive anxiety	6.92 (3.15)	6.17 (2.37)	0.024 *	0.653	0.931	0.024
Simulated F.		7.00 (2.63)	6.33 (2.19)	0.074	0.515		
Real F.	Somatic anxiety	9.33 (1.72)	9.67 (2.23)	0.776	0.082	0.832	0.061
Simulated F.		8.75 (1.71)	9.08 (1.97)	0.285	0.308		
Real F.	Self-confidence	18.67 (2.39)	19.08 (1.93)	0.180	0.387	0.221	0.354
Simulated F.		19.33 (1.23)	19.25 (1.76)	0.705	0.109		

* *p*-value < 0.05. F: Flight; CSAI-2R: Competitive State Anxiety Inventory-2R; RPE: Rating of perceived exertion; STAI: State-Trait Anxiety Inventory.

**Table 4:** HRV during simulated and real flights.

Variables	Real Flight Mean (SD)	Simulated Flight Mean (SD)	*p*-Value	Effect Size
Mean HR	93.81 (15.41)	70.83 (12.48)	0.003 *	0.847
RR	660.30 (106.86)	875.58 (139.10)	0.003 *	0.847
Pnn50	11.75 (8.83)	17.69 (14.26)	0.131	0.436
RMSSD	32.52 (14.34)	38.93 (18.04)	0.110	0.461
SDNN	53.20 (23.73)	47.98 (20.55)	1.000	<0.001
HF	20.95 (12.95)	29.06 (17.28)	0.155	0.411
LF	79.02 (12.96)	70.89 (17.27)	0.155	0.411
LF/HF	5.87 (4.11)	3.84 (2.94)	0.131	0.436
Total Power	2750.74 (1733.18)	2721.90 (2647.31)	0.929	0.026
SD1	23.02 (10.15)	27.56 (12.78)	0.110	0.462
SD2	71.14 (31.89)	61.77 (26.66)	0.657	0.128

* *p*-value < 0.05. HR: heart rate; RR: Time between intervals R-R; pNN50: Percentage of intervals >50 ms different from the previous interval; RMSSD: The square root of the mean of the squares of the successive differences of the interval RR; LF/HF: Low frequency (LF) ratio (ms2)/High frequency (HF) (ms2); Total power: The sum of all the spectra; SDNN: Standard deviation of normal-to-normal intervals; SD1: Dispersion, standard deviation, of points perpendicular to the axis of line-of-identity in the Poincaré plot; SD2: Dispersion, standard deviation, of points along the axis of line-of-identity in the Poincaré plot.

## Data Availability

Data will be available upon reasonable request to corresponding author.
